# Cartilage calcification of the ears secondary to adrenal insufficiency

**DOI:** 10.1093/skinhd/vzag006

**Published:** 2026-03-18

**Authors:** Luis Escalante, Carmen Santamaria, Astrid Maldonado, Edgar Escalante, Emma Craythorne

**Affiliations:** St John’s Institute of Dermatology, King’s College London, Guy’s and St Thomas’s Hospitals, London, UK; Department of Dermatology, King’s College London, London, UK; Department of Dermatology, Royal College of Physicians, London, UK; Department of Dermatology, Universidad Espititu Santo, Samborondon, Ecuador; Department of Dermatology, Enrique Garces Hospital, Quito, Ecuador; Department of Dermatology, Ephora Hospital, Ambato, Ecuador; Department of Dermatology, Guayaquil Universisy, Guayaquil, Ecuador; Department of Dermatology, Guayaquil Universisy, Guayaquil, Ecuador; Department of Otorhinolaryngology, Hospital Carlos Andrade Marin, Quito, Ecuador; St John’s Institute of Dermatology, King’s College London, Guy’s and St Thomas’s Hospitals, London, UK

## Abstract

Calcification of the auricular cartilage is a rare condition, associated with either local or systemic causes. Among systemic causes, endocrine disorders, particularly adrenal insufficiency, are the most frequently reported. A 41-year-old man presented with a 1-year history of stiffness and pain in both ears. The ears became swollen and erythematous and, over time, they became painful to touch or move. Physical examination revealed rigid, immobile auricles. Biopsy, X-ray and computed tomography (CT) confirmed cartilage calcification. Laboratory tests showed markedly low cortisol and adrenocorticotropic hormone levels, leading to the diagnosis of secondary adrenal insufficiency. Auricular cartilage calcification is a rare but significant manifestation of adrenal insufficiency. Skull X-rays, noncontrast CT and laboratory tests are essential for diagnosis. No effective treatment currently exists to reverse calcification. Early recognition and hormone replacement therapy can prevent further progression.

## Teaser

We present a rare case of bilateral auricular cartilage calcification secondary to adrenal insufficiency in a 41-year-old male. Diagnosis was confirmed through imaging, histopathology and hormonal assays. This case highlights the importance of considering endocrine disorders in patients with unexplained stiff and painful ears.

What is already known about this topic?

- Auricular cartilage calcification is a rare condition; it may result from local insults such as trauma, frostbite or chronic inflammation, but in some cases it reflects a systemic disorder, most notably adrenal insufficiency.
- Among endocrine-related causes, secondary adrenal insufficiency is particularly associated with bilateral auricular involvement.
- Although the clinical presentation is well described, the underlying mechanisms remain poorly understood and there is no established treatment to reverse the calcification.

What does this study add?

- This case highlights auricular cartilage calcification as an initial and isolated manifestation of secondary adrenal insufficiency in a middle-aged man, confirmed through imaging and histopathology.
- It provides a detailed discussion of the possible pathophysiological mechanisms linking cortisol deficiency to ectopic mineralization, including extracellular matrix disruption, chondrocyte transdifferentiation and impaired clearance due to cartilage avascularity.
- By integrating clinical, radiological and biochemical findings, this report reinforces the importance of considering endocrine dysfunction in patients presenting with firm, painful ears potentially enabling earlier diagnosis and intervention in cases of life-threatening hormonal deficiency.

Auricular cartilage calcification is a rare phenomenon that may be secondary to local factors such as trauma, frostbite, inflammation or systemic conditions, particularly endocrine disorders. Adrenal insufficiency, especially secondary adrenal insufficiency, is one of the most frequently reported systemic causes. Fewer than 200 cases have been documented in the literature. The elastic cartilage of the auricle and external auditory canal is typically affected, while the nasal and epiglottic cartilage is usually spared. Early management of the underlying endocrine disorder may prevent progression.

## Case report

**Figure 1:**
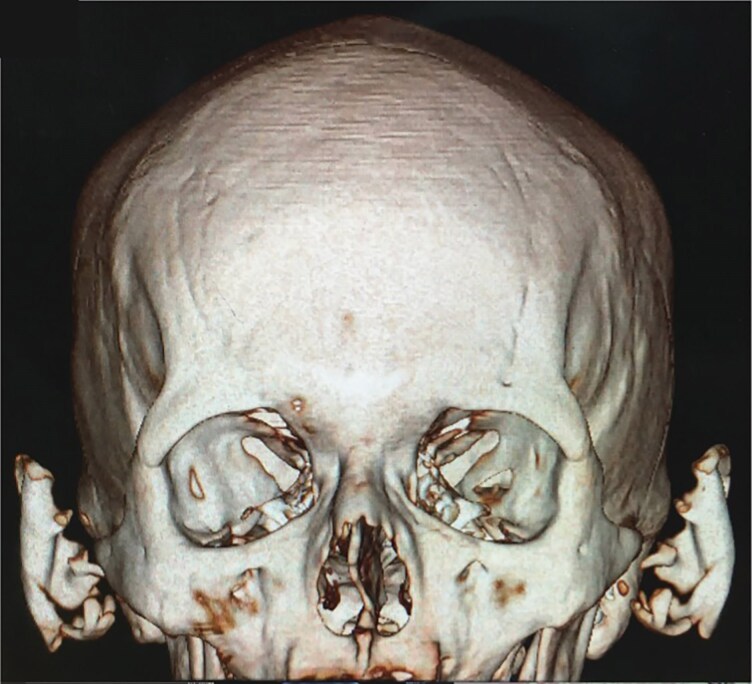
Computed tomography three dimensional reconstruction showing complete calcification of auricular cartilage in both ears.

**Figure 2:**
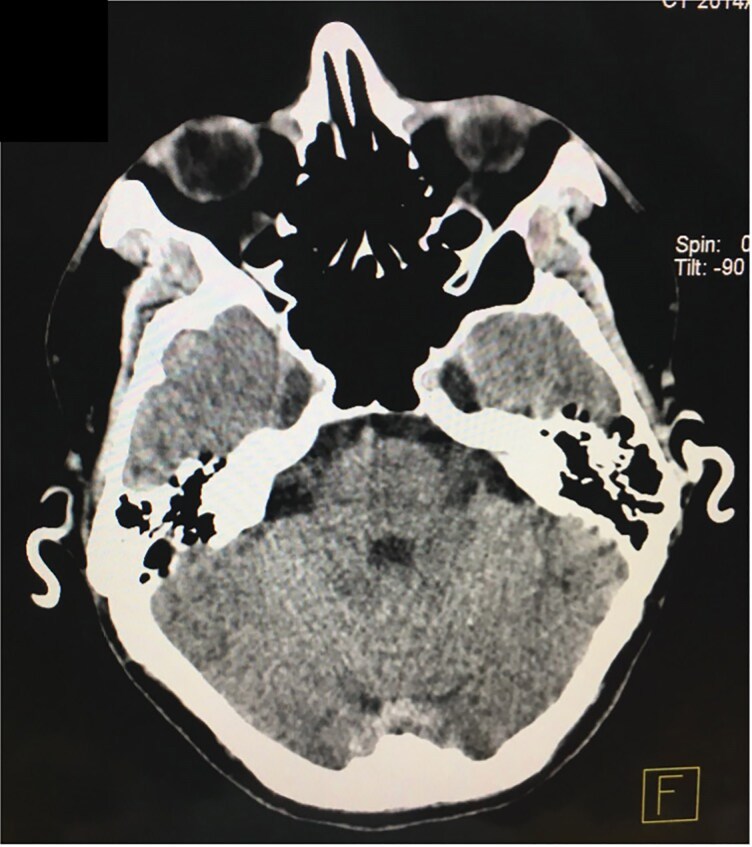
Axial computed tomography image showing bilateral auricular cartilage calcification.

**Figure 3:**
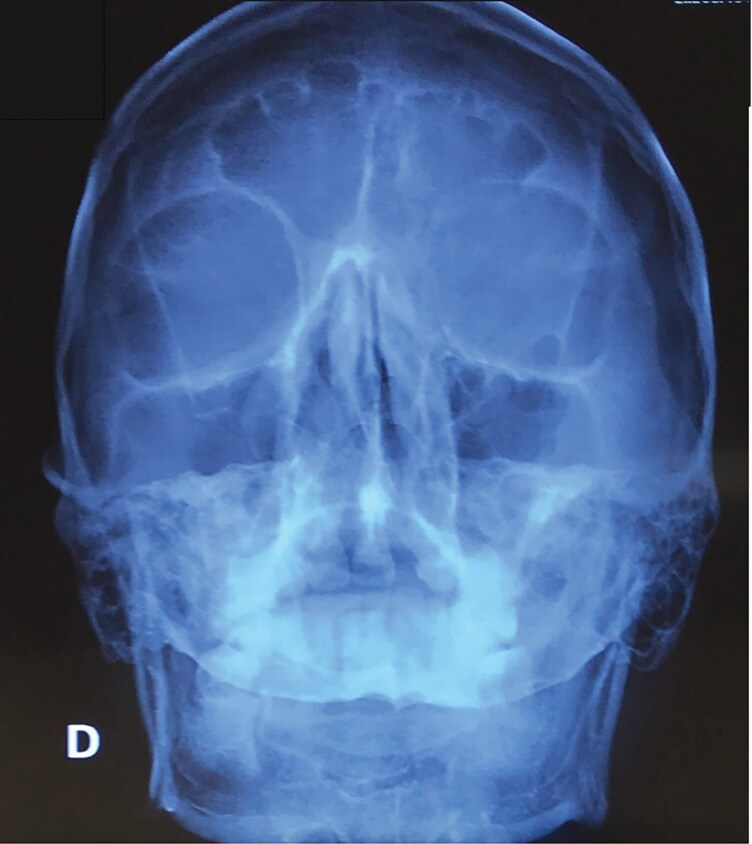
Skull X-ray showing bilateral radio-opacity in the auricular regions.

**Figure 4:**
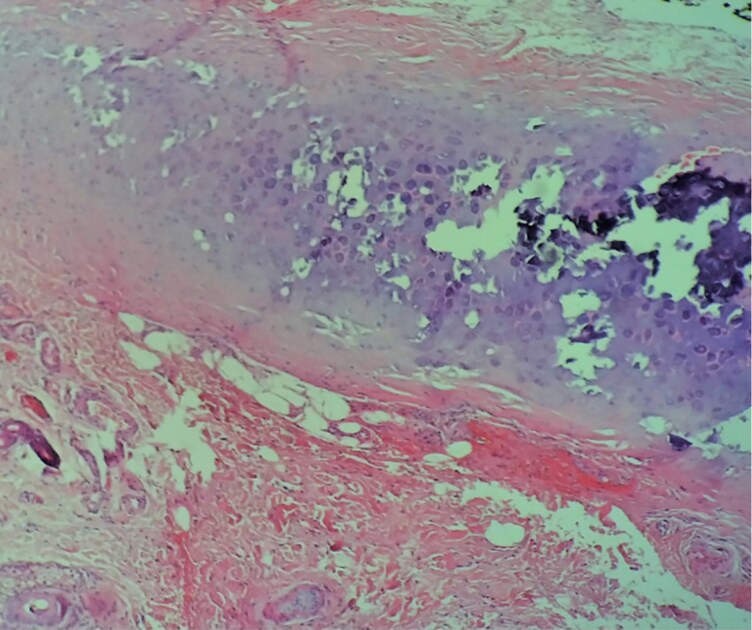
Histopathology slide showing transition from normal to calcified cartilage left to right (haematoxylin and eosin staining, original magnification ×40).

A 41-year-old man presented with a 1-year history of progressive stiffness and pain in both ears. Initially, the ears became swollen and red, followed by increasing sensitivity to touch and discomfort during sleep due to pressure. He had been treated with multiple courses of topical corticosteroids over several months without improvement. No additional systemic symptoms were reported. His past medical history included hypertension, but he was not on any regular medications. On examination, the ears appeared slightly erythematous and the auricular cartilage was extremely hard and inflexible. A skin and cartilage biopsy was performed on the right helix, which confirmed cartilage calcification. No inflammatory infiltrate, granulomas, crystals or ulceration were observed, which helped exclude relapsing polychondritis, chondrodermatitis nodularis helicis (CNH) and gout. Plain radiographs showed radio-opacity of the auricles, while noncontrast head computed tomography (CT) revealed increased density in the auricular regions consistent with calcification. Three-dimensional reconstruction demonstrated complete calcification involving the helix, antihelix, antitragus and concha of both ears. Magnetic resonance imaging of the pituitary gland revealed no structural abnormalities, with normal size, contour and signal intensity of the anterior and posterior pituitary and pituitary stalk (Figures 1–4). Laboratory analysis revealed significantly decreased morning cortisol (0.14 µg dL^–1^; reference range: 6.00–19.40 µg dL^–1^), taken at 8:15 a.m., and evening cortisol (0.8 µg dL^–1^; reference range: 2.3–11.9 µg dL^–1^). Adrenocorticotropic hormone (ACTH) was also low (4.23 ng L^–1^; reference range: 7.2–63.3 ng L^–1^). Autoimmune adrenal antibodies, including 21-hydroxylase and adrenal cortex antibodies, were negative, supporting the diagnosis of secondary adrenal insufficiency. Fasting glucose and serum electrolytes were normal. Serum uric acid was within normal limits, helping exclude gout (Table 1). Lying and standing blood pressure readings showed no orthostatic hypotension. There was no prior history of similar auricular symptoms, trauma or frostbite. Chondrodermatitis nodularis helicis was considered in the differential diagnosis, but it was deemed unlikely due to bilateral involvement and the absence of ulceration or nodular changes. Dermoscopy revealed only nonspecific erythema, with no evidence of vascular patterns, scaling or other dermatoscopic abnormalities. The patient was started on hydrocortisone replacement therapy in divided doses for the management of chronic adrenal insufficiency.

**Table 1:** Summary of laboratory results

Parameter	Result	Reference range	Interpretation
Morning cortisol (8:15 a.m.)	0.14 µg dL^–1^	6.00–19.40 µg dL^–1^	Markedly decreased
Evening cortisol	0.8 µg dL^–1^	2.3–11.9 µg dL^–1^	Decreased
Adrenocorticotropic hormone	4.23 ng L^–1^	7.2–63.3 ng L^–1^	Decreased
21-Hydroxylase antibodies	Negative	Negative	Normal
Adrenal cortex antibodies	Negative	Negative	Normal
Serum uric acid	4.5 mg dL^–1^	3.4–7.0 mg dL^–1^	Normal
Fasting glucose	93 mg dL^–1^	70–100 mg dL^–1^	Normal
Sodium	138 mmol L^–1^	135–145 mmol L^–1^	Normal
Potassium	4.1 mmol L^–1^	3.5–5.0 mmol L^–1^	Normal
Creatinine	0.8 mg dL^–1^	0.6–1.3 mg dL^–1^	Normal
Alanine aminotransferase	26 U L^–1^	<40 U L^–1^	Normal
Aspartate aminotransferase	22 U L^–1^	<40 U L^–1^	Normal
C-reactive protein	<1.0 mg L^–1^	<5.0 mg L^–1^	Normal
Erythrocyte sedimentation rate	7 mm h^–1^	0–15 mm h^–1^ (male)	Normal

## Discussion

Auricular cartilage calcification is an uncommon condition that can result from local and systemic causes. Local causes include trauma, frostbite or chronic inflammation. Systemic causes are less frequent but more clinically significant, particularly those involving endocrine dysfunction such as adrenal insufficiency. Despite being documented in fewer than 200 cases, this condition is clinically important. In most cases, the elastic cartilage of the external ear is involved, sparing other cartilage types such as the nasal septum or epiglottis. The pathophysiology behind adrenal insufficiency-induced cartilage calcification remains poorly understood.^1,2^

This condition probably results from a multifactorial process involving hormonal, cellular and mechanical factors. Cortisol deficiency disrupts cartilage homeostasis by impairing proteoglycan and collagen synthesis, weakening the extracellular matrix and facilitating mineral infiltration. In this altered environment, chondrocytes may undergo phenotypic transformation into osteoblast-like cells, expressing bone matrix proteins such as alkaline phosphatase and collagen type X. Simultaneously, increased chondrocyte apoptosis may lead to the release of matrix vesicles, which serve as nucleation sites for calcium phosphate deposition.

**Figure 5:**
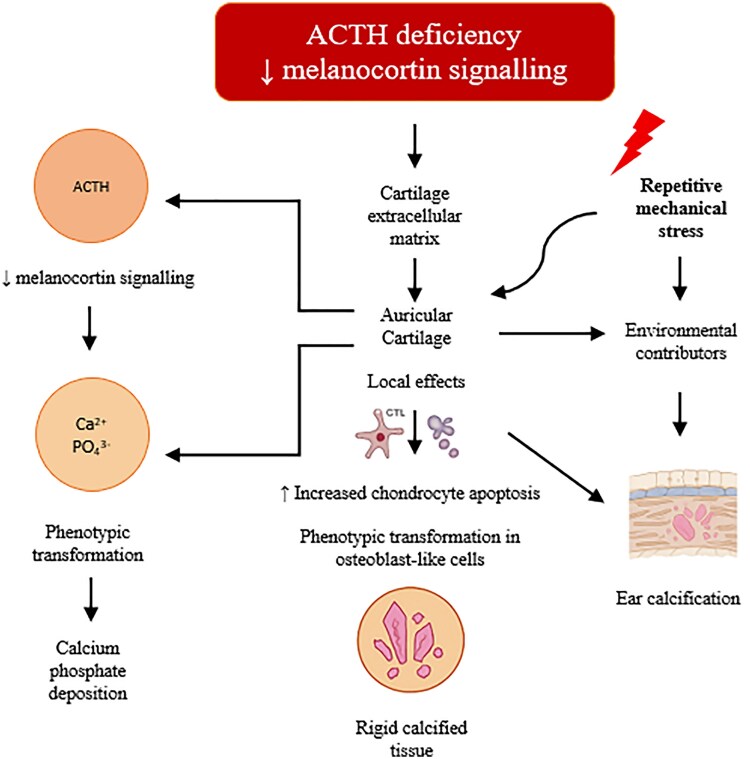
Diagram of pathophysiological mechanisms in cartilage calcification. ACTH, adrenocorticotropic hormone.

These local changes are compounded by disturbed systemic calcium–phosphate balance, reduced ACTH-mediated melanocortin signalling and the avascular nature of auricular cartilage, which limits clearance of mineral deposits. Repetitive mechanical stress further contributes to tissue injury and progressive calcification. Together, these mechanisms may explain the pathophysiological transformation of elastic cartilage into rigid, calcified tissue in the context of adrenal insufficiency (Figure 5).

Despite the combination of markedly reduced basal cortisol and ACTH levels supporting the diagnosis of adrenal insufficiency, a limitation of this report is that dynamic stimulation testing was not performed. Although the histological analysis confirmed cartilage calcification, it revealed no inflammatory infiltrate, crystal deposition, ulceration or granulomatous changes. These findings argue against relapsing polychondritis, CNH and gout. However, it is important to consider that topical corticosteroids may have attenuated any inflammatory response, potentially altering histological interpretation.

When a patient presents with firm, painful ears, auricular calcification should be considered. Diagnostic workup should include skull X-rays, noncontrast CT of the head and comprehensive laboratory investigations to evaluate for underlying endocrine or metabolic abnormalities.^3^ This is especially important as auricular calcification may be an indicator of life-threatening systemic disease. Currently, no therapies exist to reverse the calcification process.^4^ Therefore, treatment focuses on managing the underlying systemic condition to prevent further progression.

### Author contributions

Luis Escalante (Conceptualization [equal], Formal analysis [equal], Supervision [equal], Validation [equal], Writing—original draft [equal], Writing—review & editing [equal]), Carmen Santamaria (Conceptualization [equal], Data curation [equal], Formal analysis [equal], Resources [equal], Supervision [equal], Writing—original draft [equal], Writing—review & editing [equal]), Astrid Maldonado (Conceptualization [equal], Formal analysis [equal], Supervision [equal], Validation [equal], Writing—original draft [equal], Writing—review & editing [equal]), Edgar Escalante (Conceptualization [equal], Formal analysis [equal], Supervision [equal], Validation [equal], Writing—original draft [equal], Writing—review & editing [equal]), and Emma Craythorne (Supervision [equal], Validation [equal], Writing—review & editing [equal])

## References

[1] Shah A, Khorshid SM, Suchak R et al Unilateral petrified ear. Clin Exp Dermatol 2019; 44:310–12. 29947054 10.1111/ced.13698

[2] Weiss E, Degesys CA, Stroud CM. Petrified ear - a case report and review of the literature. Dermatol Online J 2017; 23:13030/qt207579b9. 28329496

[3] Taguchi T, Yoshida M, Terada Y. Petrified ear auricles with isolated adrenocorticotropic hormone deficiency. Intern Med 2017; 56:3263–4. 29021427 10.2169/internalmedicine.9108-17 PMC5742405

[4] Kannenberg S, Meyhöfer S, Lehnert H et al Petrifying: ears as hard as stone in adrenal insufficiency. Lancet Diabetes Endocrinol 2021; 9:406. 33891887 10.1016/S2213-8587(21)00112-1

